# Effects of combination of melatonin and L-carnitine on in vitro maturation in mouse oocytes: An experimental study

**DOI:** 10.18502/ijrm.v22i7.16961

**Published:** 2024-09-12

**Authors:** Raziye Chegini, Morteza Sadeghi, Sadegh Shirian, Fatemeh Sabbaghziarani, Ehsan Aali, Pouriya Soleimani, Mohammad Reza Ashtari Majelan, Fariba Zafari, Shahram Darabi

**Affiliations:** ^1^Department of Anatomical Sciences, School of Medicine, Tehran University of Medical Sciences, Tehran, Iran.; ^2^Human Genetic Research Center, Baqiyatallah University of Medical Sciences, Tehran, Iran.; ^3^Department of Pathology, School of Veterinary Medicine, Shahrekord University, Shahrekord, Iran.; ^4^Shiraz Molecular Pathology Research Center, Dr. Daneshbod Path Lab, Shiraz, Iran.; ^5^Cellular and Molecular Research Center, Research Institute for Prevention of Non-communicable Disease, Qazvin University of Medical Sciences, Qazvin, Iran.; ^6^Department of Nursing, Faculty of Nursing and Midwifery, Qazvin University of Medical Sciences, Qazvin, Iran.

**Keywords:** In vitro oocyte maturation, Melatonin, L-carnitine.

## Abstract

**Background:**

Melatonin and L-carnitine are free radical scavengers with antiapoptotic and antioxidant properties that improve oocyte development.

**Objective:**

This study aimed to find the possible effect of combining 2 antioxidant agents of melatonin and L-carnitine on oocyte morphology, maturation, apoptosis, and expression of bone morphogenetic protein 15 (*BMP-15*) and growth differentiation factor 9 (*GDF-9*) genes in a mice model.

**Materials and Methods:**

To overstimulation, 60 female NMRI mice were injected intraperitoneally using mare serum gonadotropin. On day 2 post-injection, 70 cumulus-oocyte complexes were collected from each mouse. The collected oocytes randomly were then divided into 4 groups including, the control, melatonin, L-carnitine, and melatonin + L-carnitine groups. The morphology and maturation rate of the oocytes was evaluated using a light microscope. Apoptosis was identified by terminal deoxynucleotidyl transferase-mediated dUTP nick end labeling assay and the expression of *BMP-15* and growth and differentiation factor *GDF-9* genes was also evaluated by real-time polymerase chain reaction.

**Results:**

Oocyte diameter significantly was increased in combination treatment of L-carnitine and melatonin compared to other groups (p < 0.05). L-carnitine group showed the highest mean percentage of oocyte cytoplasmic pattern. Results of the terminal deoxynucleotidyl transferase-mediated dUTP nick end labeling indicated that the lowest apoptosis rate belonged to the melatonin + L-carnitine group. Moreover, the combination groups showed the highest number of oocytes and maturation rate. The *BMP-15* and *GDF-9* genes were significantly upregulated in all treatment groups compared to the control group.

**Conclusion:**

Our results suggested a combination of melatonin + L-carnitine administration as a more effective choice for in vitro promotion of oocyte maturation.

## 1. Introduction 

In vitro maturation (IVM) is a promising technique for maturation of oocytes in the laboratory (1). The quality of oocytes using IVM techniques is suboptimal, which leads to an increase in embryo developmental abnormalities. Production of high reactive oxygen species (ROS) levels during IVM alters the function of the cultured cells and negatively affects embryo development (2). Excessive production of ROS and further reactions of these radicals with cellular molecules (i.e., proteins, lipids, and DNA) may change the functions of these molecules, causing serious problems, including DNA fragmentation, enzymatic inactivation, and ultimately cell death (3). Therefore, using oxidative homeostasis maintenance can improve the quality of oocytes and oxidative homeostasis maintenance. On the other hand, the quality of embryo development depends on factors that maintain the balance between the production of ROS and antioxidant enzymes (4).

It has also been shown that the culture media can be a source of ROS depending on its composition (5). The bone morphogenetic protein 15 (*BMP-15*) and growth differentiation factor 9 (*GDF-9*) are oocyte-secreted factors. They play an important role in controlling the ovarian function in female reproduction and act as potent regulators of folliculogenesis and ovulation (6). It has been shown that BMP-15 and GDF-9 regulate the function of granulosa cells (GCs) during follicular development. GCs has been shown to contribute to oocyte antioxidant defense against ROS (7).

Melatonin (N-acetyl-5-methoxytryptamine) is a product of the pineal gland that plays several physiological roles, including antioxidant and antiapoptotic activities. Its amphiphilic nature allows this hormone to cross biological membranes to reach subcellular compartments, thereby protecting macromolecules against the deleterious effects of oxidative stress. Oxidant/antioxidant balance within follicles seems crucial for normal oocytes and normal functions of GCs. Accumulating evidence has indicated that melatonin protects these cells from oxidative stress. Based on its concentration in the culture medium, melatonin has been shown to have positive or deleterious effects on embryo development. It can regulate the balance between antioxidant and pro-oxidant activity (8). Melatonin improves oocyte quality by influencing the expression of genes linked to oocyte maturation (such as *BMP-15* and *GDF-9*) in polycystic ovarian syndrome. High melatonin concentrations are thought to offer important support during the IVM process. L-carnitine has also been suggested to be used as a novel noninvasive factor to improve not only oocyte quality also embryonic development due to its antioxidant and antiapoptotic effects (9). It was showed that L-carnitine as a strong supplement has been demonstrated to eliminate the cyclophosphamide induced-destructive and harmful effects during chemotherapy in cancer patients and were significantly upregulated by expression levels of oocyte quality genes, including *GDF-9*. Although studies have reported the antioxidant and antiapoptotic effects of melatonin and L-carnitine alone in oocyte culture environments (9, 10), the combination of melatonin and L-carnitine effects on the quality of embryo development and IVM, as well as apoptosis has yet therefore, the present study investigated the effects of supplementing the IVM culture medium with antioxidants on morphology, maturation rate, and apoptosis of the oocytes, as well as *BMP-15* and *GDF-9* gene expression in the mice model.

## 2. Materials and Methods

### Animals

A total of 60 female NMRI mice (4–6 wk, 25–30 gr) were obtained from the anima house of Qazvin University of Medical Sciences, Qazvin, Iran. Mice were kept in standard condition (22ºC, 12 hr light/dark cycle).

### Superovulation and oocyte collection

Animals were intraperitoneally injected with 7/5 IU pregnant mare serum gonadotropin (PMSG, Millipore, USA) for superovulation. Over a period of 48 hr postinjection of PMSG, the animals were euthanized. The ovaries were dissected and placed in 1 ml of warm minimum essential medium (MEM) alpha (αMEM) (Gibco, USA). Approximately 70 cumulus-oocyte complexes (COCs) were obtained from each mouse. The COCs were removed from oocytes using an insulin syringe needle under an inverted microscope (Optika, XDS-2, Italy).

### IVM supplemented with melatonin and L-carnitine supplementation

After collection, immature COCs (cumulus thickness, compactness granulation, and homogeneity of the ooplasm) were washed 3 times in αMEM, placed in a drop of mineral oil, and incubated at 37 C and 5% CO ${\;}_{2}$ in base culture medium. αMEM supplemented with 5% fetal bovine serum (Invitrogen, USA), 10% human chorionic gonadotropin (Sigma, Germany), 2 µmol fibroblast growth factor, and epidermal growth factor (Peprotech, USA) were used as a base culture medium (11). For experimental groups, the harvested oocytes were randomly divided into 4 groups and assessed for maturation, morphology, and apoptosis. In the control group (I) COCs were cultured without any antioxidant interaction in base culture medium. In the melatonin alone group (II) the base culture medium was supplemented with 2 µmol melatonin (Sigma, Germany) (12). In the L-carnitine alone group (III) the base culture medium was supplemented with 0.6 mg/ml L-carnitine (Sigma, Germany) (13). In the melatonin + L-carnitine group (IV) the base culture medium was supplemented with a combination of 0.6 mg/ml L-carnitine plus 2 µmol melatonin. The number of oocytes evaluated in control, melatonin alone, L-carnitine alone, and melatonin + L-carnitine groups was 220, 200, 190, and 245, respectively. After 24 hr, metaphase II (MII) oocytes were identified with the extrusion of polar body (PB) 1, and maturation rates of each group were recorded.

### Total oocyte score (TOS)

The harvested oocytes from all experimental groups were assessed based on the TOS with 6 criteria for oocyte quality, including size, morphology, cytoplasm, zona pellucida (ZP), perivitelline space (PVS), and PB. 3 values were assigned to each parameter: -1 (worst), 0 (average), or +1 (best) (14) (Table I).

### Oocyte maturation rate and terminal deoxynucleotidyl transferase-mediated dUTP nick end labeling (TUNEL) assay

Oocyte maturation, a unique type of cell division, is different from mitosis. An oocyte is arrested at meiotic prophase I, which contains a large nucleus covered by a nuclear envelope known as the germinal vesicle. Then, following a spike in luteinizing hormone, chromosome condensation gradually happens because of germinal vesicle breakdown. The oocyte enters the metaphase I stage when the chromosomes are completely condensed. In order to prepare for chromosome segregation in the ensuing anaphase I, spindle microtubules attach to the kinetochores and chromosomes align on the metaphase plate during this stage. Oocytes stop at MII after the first PB is excluded. Therefore, the maturation rate of the oocytes was determined based on the extrusion of the first PB. The morphology of all the oocytes was then investigated using an inverted microscope (Optika, XDS-2, Italy) pre- and post-IVM. Images of the oocytes were acquired using a digital camera (Delta Pix, USA). After several washing steps with phosphate-buffered saline (Gibco, USA), all the matured oocytes were fixed in 4% paraformaldehyde (Wako, Japan) to measure the apoptosis rate using TUNEL assay and propidium iodide staining. All the cells were covered with H ${\;}_{2}$ O ${\;}_{2}$ (Sigma, Germany) twice every 10 min. Oocytes were then incubated with proteinase K for 30 min followed by 10 min incubation in 0.3% Triton X-100 (Sigma, Germany) before incubation in the TUNEL solution (Roche, Germany) for 2 hr at 37 C, according to the manufacturer's instructions. Oocytes of the negative control group were only incubated with a fluorescent solution without exposure to the terminal deoxynucleotidyl transferase enzyme to ensure no labeling activity occurred. Oocytes of the positive control group were incubated with 50 µg/ml DNase I solution (Sigma, Germany) for 1 hr before incubation with TUNEL staining solution. To label the nuclei, all the oocytes were stained with 50 µg/ml propidium iodide (Sigma, Germany) solution for 10 min and considered under a fluorescent microscope (Labomed, USA).

### Real-time polymerase chain reaction (PCR)

#### RNA extraction and cDNA synthesis 

Total ribonucleic acid (RNA) was extracted from the MII oocytes (n = 10 in each group in 3 repeats) using RNeasy Mini kit (Qiagen, Germany) according to the manufacturer's instructions. The purity of extracted RNAs was qualified using NanoDrop (Thermo Fisher Scientific, USA). For mRNA expression analysis, the extracted RNA was reverse transcribed to cDNA using QuantiNova Reverse Transcription (Qiagen, Germany).

The cDNA was amplified using TB Green Premix Ex Taq II (Takara, Japan), and gene expression was determined with a real-time PCR (Corbett Rotor-Gene 6000). β*-actin* was used as internal control. Each sample was done in triplicate. The quantitative RT-PCR cycle consisted of 95 C for 2 min followed by 45 cycles of amplification (95 C, 59 C, and 95 C for sec, 30 sec, and 1 min, respectively). The relative quantification of *GDF-9* and *BMP-15* gene expression was analyzed using StepOne software V2.0 (Fisher Scientific, Loughborough, UK) and compared to the β*-actin* gene and analyzed using the 2- Δ Δ CT method. The primer sets' sequences were selected from NCBI (Table II).

**Table 1:** Total oocyte scoring (TOS)


[1.2in,lr]**Parameter** **Score**	**+1**	**0**	**-1**
**Oocyte shape**	Judged to be normal	Almost normal (less dark and/or less ovoid shape)	Poor (dark general and/or ovoid shape)
**Oocyte size**	> 60 μ and < 100 μ	Did not deviate from normal by more than 10 μ	Below 50 μ or greater than 100 μ
**Ooplasm characteristics**	Absence of granularity and inclusions	Slightly granular and/or only a few inclusions	Ooplasm was very granular and/or very vacuolated and/or several inclusions
**PVS**	Normal-size PVS with no granules	Moderately enlarged PVS and/or small PVS and/or a less granular PVS	An abnormally large PVS, an absent PVS, or a very granular PVS
**ZP**	> 4.3 μ and < 8.1 μ	Did not deviate from normal by more than 1 μ	Deviate from normal by more than 1 μ
**PB morphology**	Normal size	Judged a fair but not excellent	Flat and/or multiple PBs, granular and/or either abnormally small or large PBs
PVS: Perivitelline space, ZP: Zona pellucida, PB: Polar body			

**Table 2:** The sequences of the used primers in the current study


**Gene **	**Primers**
***GDF-9***	5 ' -CCTTCTTAGTTCTTCCAAGTCATG 5 ' -CTAACGACAGGTGCACCTCGT
***BMP-15***	5-AGCAACCAGGTGACAGGA-3 5-CCTCCTTTACCAGGTCA-3
**β** ***-actin***	5 ' -TCGTGGGCCGCTCTAGGCAC-3 ' 5 ' -TGGCCTTAGGGTTCAGGGGG-3 '
*GDF-9*: Growth differentiation factor 9, *BMP-15*: Bone morphogenetic protein 15	

### Ethical considerations

This experimental study was conducted according to the guidelines of Qazvin University of Medical Sciences for Animal Care and approved by the Ethics Committee at Qazvin University of Medical Science, Qazvin, Iran (Code: IR.QUMS.REC.1398.165).

### Statistical analysis

All the data were analyzed using Statistical Package for the Social Sciences, version 23.0 (IBM, SPSS Inc, Chicago, USA). The one-way ANOVA followed by post hoc Tukey and Chi-square tests were used to analyze the comparison of gene expression and morphology, respectively. P ≤ 0.05 was considered statistically significant.

## 3. Results 

### TOS findings

**Figure 1:**
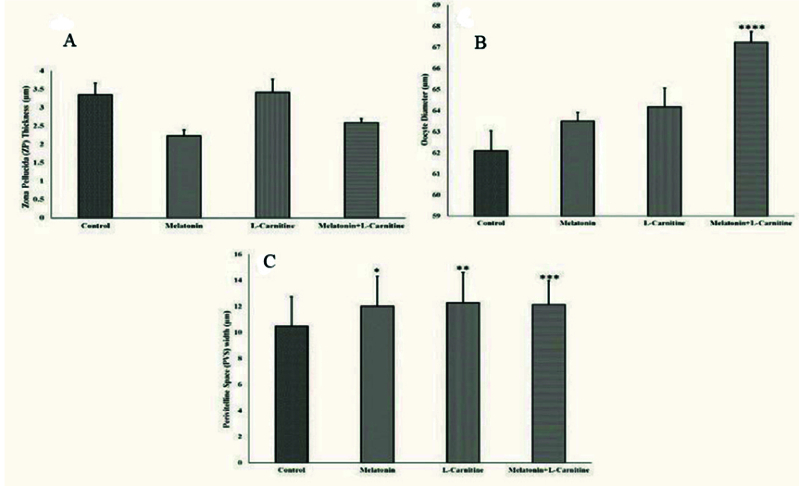
Scoring parameters of oocytes in different groups. A) Zona pellucida, B) Oocyte diameter, and C) Perivitelline space. *p = 0.02, ** and ***p ≤ 0.001, and ****p = 0.003 vs control.

No significant differences in ZP thickness were observed between different groups in this study. The ZP thickness of the oocytes of the melatonin + L-carnitine and control groups was 2.58 ± 0.12 µm and 3.34 ± 0.32 µm, respectively (Figure 1a). The oocyte diameter was significantly increased to 67.24 ± 0.5 µm in the melatonin + L-carnitine group compared to the control group with the oocyte diameter of 62.09 ± 0.95 µm (p = 0.003). Oocyte diameter showed no significant change in the melatonin (63.49 ± 0.42) and L-carnitine (64.16 ± 0.9) alone groups compared to the control group (Figure 1b). The PVS was significantly expanded in all treatment groups compared to the control group (the melatonin alone, p = 0.02; and L-carnitine alone, p < 0.001; and melatonin + L-carnitine groups, p < 0.001). However, these changes were not statistically significant between the treatment groups (Figure 1c).

**Figure 2:**
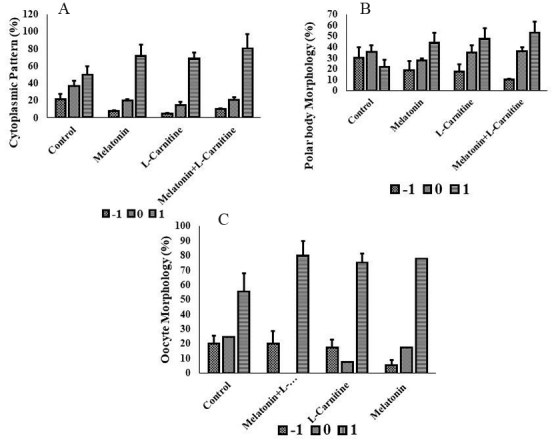
Scoring parameters of oocytes in different groups. A) Cytoplasm grading, B) Polar body, and C) Oocyte morphology. In all the treated groups, most of the oocytes fitted grade +1 compared to the control group.

The cytoplasmic pattern of most of the oocytes in the melatonin, L-carnitine, and melatonin + L-carnitine groups fitted the +1 grade compared to control group; 72 ± 12.8%, 69 ± 17%, and 80 ± 6.5%, respectively. The highest mean percentage of +1 graded cytoplasmic pattern belonged to the melatonin + L-carnitine group (Figure 2a). As compared to the control group, most of the PBs in the melatonin, L-carnitine, and melatonin + L-carnitine groups were in the +1 grade. The mean percentages of +1 graded PBs in the melatonin, L-carnitine, and melatonin + L-carnitine groups were 44 ± 12%, 47.5 ± 9.8%, and 53.4 ± 14%, respectively (Figure 2b). The mean percentage of the oocyte morphology with grade +1 was higher in the treatment groups than in the control group. The melatonin + L-carnitine group presented the highest mean percentage of oocytes with a +1 grade. These percentages for melatonin, L-carnitine, and melatonin + L-carnitine groups were 77.6 ± 10.3%, 75 ± 6.5%, and 80 ± 9.8%, respectively (Figure 2c).

### Oocyte maturation rate and TUNEL assay

**Figure 3:**
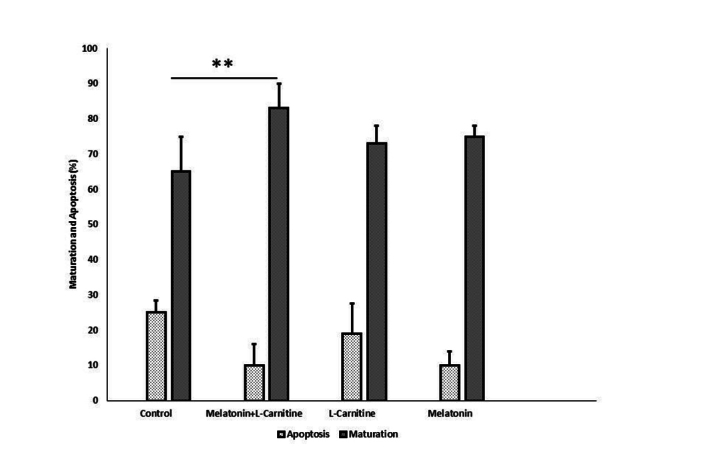
The graph shows the comparison of the maturation rate of the oocytes (Oocytes [MII] rate was determined based on the extrusion of the first PB) and apoptosis rate. The rate of apoptosis in the treated groups was lower than the control. **p < 0.01 vs control.

**Figure 4:**
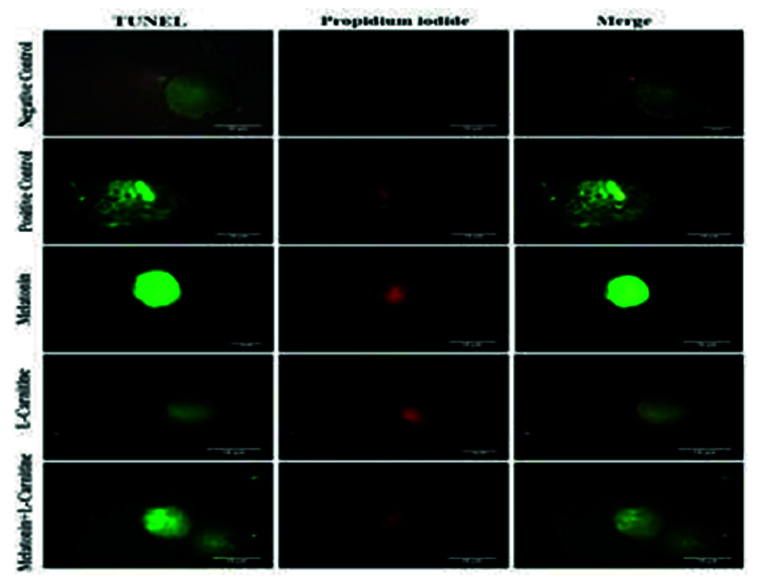
DNA fragmentation in oocytes was assessed using the TUNEL assay and propidium-iodide staining. Negative control oocytes were incubated only in the fluorescent solution lacking the enzyme to ensure the absence of labeling. Regarding the positive control, several oocytes were treated with the DNase I solution and then the TUNEL solution. Under a fluorescent microscope, oocytes were stained with a propidium iodide solution to identify their nuclei. All images are shown at x400 magnification.

The oocyte maturation rate was significantly increased in the treatment groups compared to the control group. The highest maturation rate was detected in the melatonin + L-carnitine group (83 ± 6%). The mean percentage of maturation rate in the L-carnitine alone and melatonin alone groups was 73 ± 5.2% and 75 ± 4%, respectively. The apoptosis rate was assessed using the TUNEL assay, and it was reduced in the treatment groups compared to control group. Amongst the treatment groups, the lowest apoptosis rate was seen in the melatonin + L-carnitine group (10 ± 5%). The rate of apoptosis in the L-carnitine alone and melatonin alone groups were 19 ± 6.1% and 16 ± 3%, respectively (Figure 3). The microscopic images of TUNEL positive and TUNEL negative oocytes in different groups are depicted in figure 4.

### *BMP-15* and *GDF-9* genes expression

**Figure 5:**
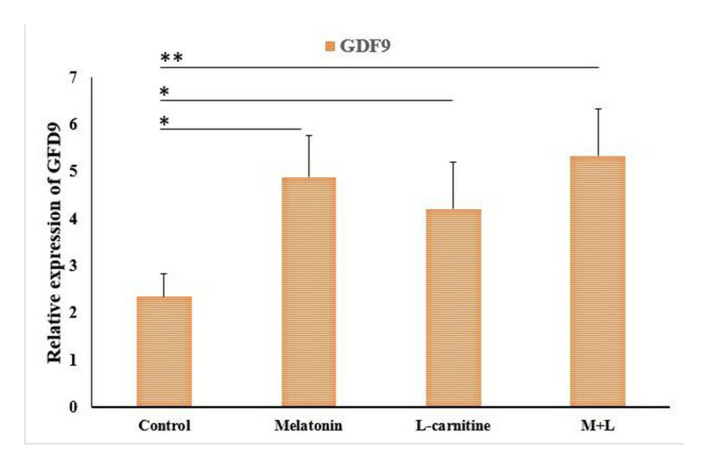
The related gene expression of *GDF-9* in various groups. ** and *indicate p < 0.001 and p < 0.01, respectively. GDF-9: growth differentiation factor 9, MDA: Melatonin, L: L-carnitine.

**Figure 6:**
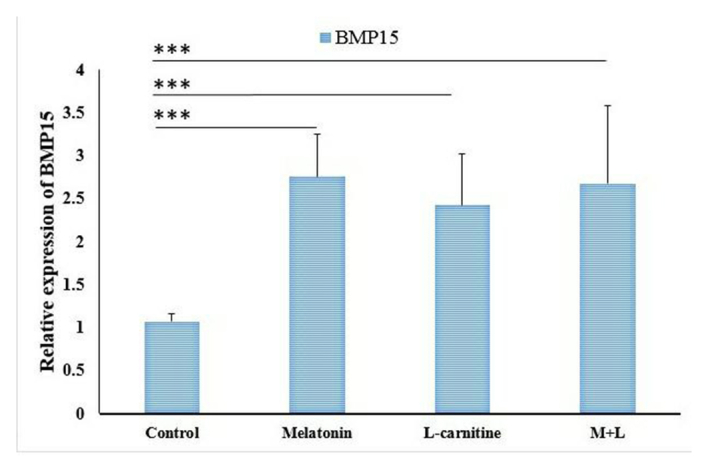
The related gene expression of *BMP-15* in various groups. *** *p* < 0.001, MDA: Melatonin, L: L-carnitine.

The gene expression levels of *BMP-15* and *GDF-9* were significantly upregulated in the combination of melatonin and L-carnitine group as well as the melatonin and L-carnitine alone groups compared to the control group (Figure 5). However, the expression levels of both genes showed no significant difference between any 3 treatment groups (Figure 6).

## 4. Discussion

This study aimed to investigate the effects of supplementing the IVM culture medium with antioxidants on oocyte quality, maturation rate, and apoptosis index of the oocytes, as well as the expression levels of *GDF-9* and *BMP-15*. Oocyte quality is one of the critical factors in embryonic development, IVM, and subsequent female fertility (15). Oxidative homeostasis has been shown to be effective for the quality of oocytes (16). Supplementing the IVM with melatonin has been shown to decrease the ROS level and inhibit apoptotic events in bovine, porcine, and human oocytes resulting in increasing their developmental potential (8, 17). It has also been demonstrated that L-carnitine supplementation during IVM of immature oocytes has also been demonstrated to improve the preimplantation developmental competence of oocytes due to cytoplasmic acceleration and nuclear maturation of oocytes (9). Supplementing the IVM with L-carnitine has been widely studied to increase the quality and maturity of oocytes in several mammalian species. Although the relationship between oocyte quality and the oocyte culture environments containing antioxidant and anti-apoptotic agents has been widely studied, the effects of the combination of melatonin and L-carnitine have not been studied. The results of this study indicated that both the oocyte quality and maturation were improved, and the apoptosis rate was decreased in all the treatment groups, especially in the melatonin + L-carnitine groups and melatonin alone, compared to the control group. Individual oocytes were assessed based on TOS with 6 parameters, including size, morphology, cytoplasm, ZP, PVS, and PB. These 6 parameters are directly predictive of embryo quality (18). The results of gene expression showed that both *BMP-15* and *GDF-9* were significantly upregulated in all treatment groups compared to the control group. Both *BMP-15* and *GDF-9* have been demonstrated to affect folliculogenesis and oocyte quality. *GDF-9* contributes to follicular development and differentiation of GCs in mice. *BMP-15* has been shown to promote follicle maturation, regulate granulosa sensitivity to follicle-stimulating hormone action, increase oocyte developmental competence, and prevent granulosa apoptosis (19). The mRNAs of BMP-15 and GDF-9 proteins have been shown to express in female mice oocytes during folliculogenesis (20).

The present study's findings demonstrated no significant difference between the control and treatment groups in terms of oocyte diameter and PVS. It has been previously reported that melatonin cannot improve cumulus expansion and PVS formation (21). We have found that both melatonin and L-carnitine alone and in combination with melatonin, cannot alter oocyte diameter and PVS in IVM. To our knowledge, there is no study about the effect of L-carnitine alone and in combination with melatonin on oocyte diameter and PVS. All treatment groups oocyte morphology, cytoplasmic pattern, and PB morphology were predominantly graded as +1. Previous studies have shown that oocytes derived from IVM have reduced developmental potential compared to those matured in vivo (22).

Moreover, poor oocyte quality plays a key role in female infertility (23). Oxidative stress is a phenomenon that occurs during the in vitro culture of oocytes since these oocytes are exposed to higher O ${\;}_{2}$ concentrations than those developing in vivo. Therefore, this increased oxidative stress is a burden for oocyte IVM. Consequently, application of antioxidants in these procedures seems to be essential (24).

L-carnitine is involved in fatty acid transportation into mitochondria for ATP synthesis via beta-oxidation. L-carnitine improves oocyte quality and early embryo development by improving mitochondrial beta-oxidation (10). Oocytes do not produce L-carnitine during IVM (18), however, its metabolite has been found within follicular fluid (22). Despite its role as a radical scavenger (25) and a potent antioxidant (22), the addition of L-carnitine to the IVM medium of bovine oocytes had no significant effect on the development potential of the embryos (26).

The obtained results of the TUNEL assay indicated that adding L-carnitine and melatonin alone or in combination with each other to the IVM culture medium significantly reduced the apoptosis rate of oocytes compared to the control group, especially the melatonin + L-carnitine. These findings may result from reducing the ROS level and inhibiting apoptotic events of melatonin and L-carnitine (27). Moreover, in contrast with these results, it has been shown that L-carnitine does not affect DNA fragmentation (26).

A significant promotion in oocyte maturity using exposure to melatonin and L-carnitine alone and in combination compared to the control group was reported in the present study. The melatonin + L-carnitine group detected the highest maturity rate. It has been reported that melatonin improves the cytoplasmic maturity of oocytes (17) in which it induces overexpression of maturation-related genes, *GDF-9*, and meiosis regulator and mRNA stability factor 1 (26), redistribution of organelles, and expression of antioxidant enzymes (17). Melatonin induces meiosis resumption, and oocytes treated with this compound are supported for subsequent development into an embryo (28). Melatonin enhances the quality of conditions required for embryo development. This is highly dependent on melatonin dosage, which, at low concentrations, may not be effective for improving embryo development, and at high concentrations, it may cause embryo damage; therefore, choosing a proper dosage for this compound is important. The reduction of ROS levels and apoptosis rate by melatonin favors promoting oocyte maturation. Melatonin, through its membrane receptors, can affect oocyte maturation. Melatonin membrane receptors, melatonin membrane receptor 1 and melatonin membrane receptor 2 are reported to be expressed in bovine oocytes (26). However, there is no similar study on the effect of L-carnitine on oocyte maturation.

In this study, the improvement of oocyte quality, maturation, and apoptosis rate in all treatment groups was consistent with the results of previous studies, which can be done through the antioxidant effects of melatonin and L-carnitine.

## 5. Conclusion

Since the findings of the present study clearly and strongly indicate that combination therapy with melatonin and L-carnitine beneficially affects oocyte quality. Considering the higher efficacy of applying melatonin + L-carnitine compared to melatonin and L-carnitine alone, it would be presumed that the application of a combination of melatonin and L-carnitine, favorably results in the promotion of oocyte quality and maturation rate. However, further research is still required for the in vitro promotion of oocyte maturation.

##  Data availability

Data supporting the findings of this study are available upon reasonable request from the corresponding author.

##  Author contributions

Raziye Chegini: Writing original draft, data collection, conceptualization. Morteza Sadeghi: Data curation, project administration. Sadegh Shirian: Writing, statistics. Fatemeh Sabbaghziarani: Drafting article. Ehsan Aali: Investigation, data analysis. Pouriya Soleimani: Data collection. Mohammad Reza Ashtari Majelan: Resources, data collection. Shahram Darabi: Writing, software. Fariba Zafari: Supervision, project administration.

##  Conflict of Interest

The authors declared no potential conflict of interest with respect to the research, authorship, and/or publication of this article.
